# Ultrasound Cine Loop Standard Operating Procedure for Benign Thyroid Diseases—Evaluation of Non-Physician Application

**DOI:** 10.3390/diagnostics11010067

**Published:** 2021-01-04

**Authors:** Philipp Seifert, Ivonne Maikowski, Thomas Winkens, Christian Kühnel, Falk Gühne, Robert Drescher, Martin Freesmeyer

**Affiliations:** Clinic of Nuclear Medicine, Jena University Hospital, Am Klinikum 1, 07747 Jena, Germany; philipp.seifert@med.uni-jena.de (P.S.); ivonne.maikowski@med.uni-jena.de (I.M.); thomas.winkens@med.uni-jena.de (T.W.); christian.kuehnel@med.uni-jena.de (C.K.); falk.guehne@med.uni-jena.de (F.G.); robert.drescher@med.uni-jena.de (R.D.)

**Keywords:** thyroid, ultrasound, cine loop, MTA, second reading, volumetric determinations, TIRADS

## Abstract

Conventional ultrasound (US) is time-consuming, and results are subjected to high interobserver variability. In this study, the reliability of a novel thyroid US cine loop standard operating procedure (SOP) applied by non-physicians (Medical Technical Assistant, MTA) is investigated. Thirty-three consecutive patients (22 females, 11 males) were enrolled. Patients underwent conventional thyroid US performed by a nuclear medicine physician and additional MTA US cine loop according to a local SOP that includes transversal and sagittal cine loops covering the entire thyroid. The video sequences were transferred to the Picture Archiving and Communication System (PACS) for second reading purposes. MTA US data were not considered for medical reports but for blinded second reading review of the PACS images. The results of conventional physician US reports and reviewed MTA US cine loops were compared regarding size determinations of the thyroid and its nodules, as well as Thyroid Imaging Reporting and Data Systems (TIRADS) classification of all identified lesions. The results revealed very high concordance between conventional physician US and MTA US cine loop review for both size measurements and TIRADS classifications (r_(s)_ = 0.84–0.99, *p* < 0.0001 each). Minor technical impairments were identified. The evaluated thyroid US cine loop SOP enables reliable second reading results and can be applied by non-physicians.

## 1. Introduction

Ultrasound (US) remains an indispensable diagnostic tool for the assessment of thyroid disease [1,2]. The method allows for detailed morphological estimation of organ structures. Pathologies like thyroid nodules (TN), organ enlargements, cysts, inflammatory infiltrations, and immunological diseases can be identified, depicted in detail, and observed over long-term treatment courses [3,4]. The importance of the method is reflected by its clinical entrenchment. A correct ultrasonographic determination of the thyroid volume is crucial for the calculation of the intended I-131 activity in preparation of a radioiodine therapy [5]. US-based risk stratification systems for TN, e.g., Thyroid Imaging Reporting and Data Systems (TIRADS) are gaining in importance and are increasingly recommended by experts and guidelines [6,7,8,9]. Complimentary US applications such as duplex sonography and elastography enable the examiner to assess blood-flow and stiffness of the organ and its lesions [10,11].

Well-known general advantages of US examinations in human medicine are its widespread availability, almost missing side effects, high level of acceptance by both healthcare professionals and patients, excellent resolution and contrast in soft tissue, real-time applicability, de facto unlimited repeatability, portability, and very low procedural costs (except for the initial purchase of a suitable device), to name only the most important [12,13,14,15].

However, one of the main drawbacks of conventional US is its examiner dependency. Significant interobserver variability has been described both for volumetric determinations and for TN risk stratification systems [16,17]. An important principle is a fact that the results of US investigations are still often only documented by static image captures only. The US operator decides which findings are worth documenting. Consequently, it is impossible to retrospectively ascertain whether the correct part of a pathological finding has been recorded or whether crucial aspects are missing. A comprehensive second reading review, as is customary with CT or MRI examinations, is therefore restricted.

Nearly all modern US devices provide an implemented video recording function that enables the operator to capture so-called cine loops and transfer them to the local Picture Archiving and Communication System (PACS). The first concepts were already described in the 1990s [18]. However, so far, this method has only been established in cardiology [19]. The application and documentation of US cine loops in other specialties are scarce, and standardized protocols are lacking in clinical practice and in guidelines. Only a few standard operating procedures (SOP) have been introduced, e.g., for abdominal US [20,21]. To the best of our knowledge, no US cine loop SOP has yet been described for benign thyroid diseases.

A second major disadvantage of US examinations is the time-consumption for the operator. Non-physician US has been proven reliable in several studies, even in emergency medicine [22,23]. In the USA, efforts are intensifying to integrate US examinations into the physician assistant curriculum [24,25]. However, the physician assistant system is a rarity outside of the United States. In Germany, for example, nurses and medical, technical assistants (MTA) account for the majority of non-physician healthcare professionals.

The aim of this study is to introduce a novel US cine loop SOP for benign thyroid diseases that can be applied by both physicians and non-physicians (in this case, MTA) and to evaluate its reliability for the purpose of a second reading review with a focus on size measurements and TIRADS classifications.

## 2. Materials and Methods

### 2.1. Patients and Ethics

All patients included in this study were referred to our university nuclear medicine department for clarification of thyroid abnormalities between February 2015 and July 2017. If conventional diagnostics (anamnesis, laboratory parameters, ultrasound, and Tc-99m-scintigraphy) revealed ambiguous findings, e.g., uncertain functional assessment of thyroid nodules, additional I-124-PET/CT, and US fusion imaging investigations were performed within the scope of several clinical study protocols (not part of the present study). The results were published [26,27,28]. A part of this patient cohort was consecutively enrolled for further US examinations performed by a MTA between July 2015 and February 2016 (data of the present study). Accordingly, the present study is part of a comprehensive perspective research project for which approval from the responsible local ethics committee has been obtained (Reference number: 4286-12/14). All patients gave their informed consent in writing. All examinations were carried out in compliance with the Declaration of Helsinki.

### 2.2. Investigators and Observers

Conventional thyroid diagnostics was performed and assessed by three experienced nuclear medicine physicians. At the beginning of the investigation period, M.F. had 20 years, T.W. and P.S. each had four years of professional experience in thyroid imaging. One of these physicians decided whether additional investigations were necessary after an immediate review of the conventional diagnostics. All results were then analyzed, a medical report was written, and a treatment course was suggested.

Independently of these results, a single MTA performed additional US examinations on the same day as the conventional US scan. The MTA (I.M.) had five years of professional experience without any US education. In the preparation of this study, a one-hour introductory US training was conducted.

### 2.3. Examination Protocols and Devices

Both conventional physician US and MTA US cine loop were performed as high-resolution B-mode US with the same US device equipped with the same linear ML6-15 probe (GE LOGIQ E9, GE Healthcare, Milwaukee, WI, USA). Patients were examined in supine position with overflexion of the head. US gel was applied. Virtual convex, crossbeam, and contrast-harmonic-imaging were turned on. Three foci were set when coverage of an entire thyroid lobe was intended, two when a nodule was of interest. The focus positions were chosen appropriately. Further parameters such as frequency (usually between 10–12 MHz), brightness, gain, zoom, and depth were individually adjusted according to the respective findings in order to achieve optimal image quality. The patients were asked to hold their breath during image acquisition.

Within the course of initial conventional diagnostics, standard thyroid B-mode US was performed by one of the above-mentioned physicians. The examinations contained measurements of the respective lobe diameters (cranial-caudal, anterior-posterior, orthograde medial-lateral) and the documentation of all identifiable TN (>5 mm) inclusive of their diameters and their Kwak–TIRADS classification [29].

**Figure 1:**
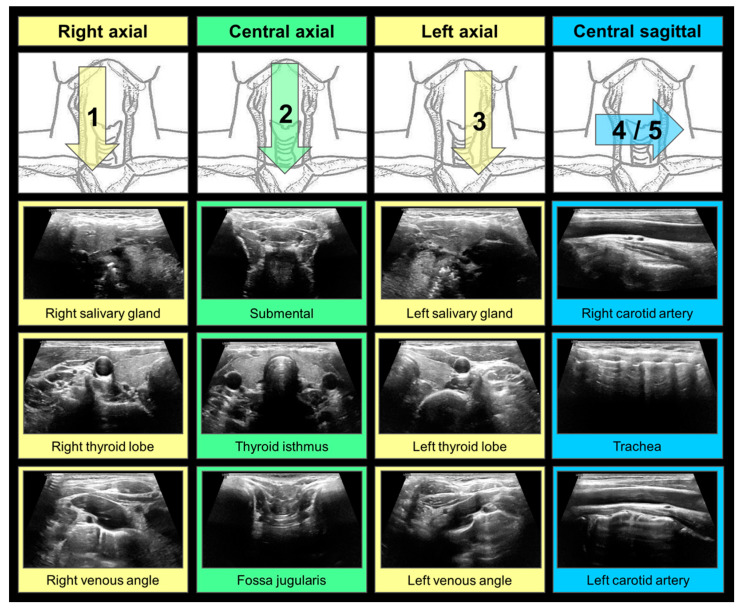
Ultrasound (US) cine loop standard operating procedure (SOP) for benign thyroid diseases. A total of four to five cine loops should be acquired. (1,3): Axial scans of the right/left cervical compartment for the depiction of the right/left thyroid lobe, extending from the submandibular salivatory glands (cranial reference organs, starting point) to the brachiocephalic venous angles (caudal reference structures, endpoint). (2): Axial scan of the central cervical compartment for the depiction of the thyroid isthmus, extending from submental (cranial reference structure, starting point) to the jugulum (caudal reference structure, endpoint). (4/5): Sagittal scans of the central cervical compartment covering the whole extent of both thyroid lobes and the isthmus, extending from the right carotid artery (right lateral reference structure, staring point) via the trachea (median reference structure) to the left carotid artery (left lateral reference structure, endpoint); depending on the shape of the larynx, two separate scans may be favorable.

The MTA exclusively recorded thyroid cine loops according to a local SOP (Figure 1). This protocol enabled the investigator to capture the entire thyroid gland as well as its surroundings in transversal and sagittal orientation within short video sequences. The cine loops were transferred to the local PACS without consultation of a physician. Neither static image captures nor any measurements were carried out. The acquired images were not taken into account for medical reports.

The authors recommended to perform the cine loop acquisition slowly (approximately 10 s per loop) to avoid blurred images, and 200–250 frames per cine loop are favorable. The entire procedure can be carried out in less than 1 min.

Five years later, in April 2020, the MTA US cine loops were reviewed on PACS by one of the above-mentioned physicians (second reading). The reviewer was a different person than the one who conducted the initial conventional US and was blinded to the medical reports. The reviewer intended to achieve the same parameters as on conventional physician US (see above).

I-124-PET/CT examinations have not been conducted for the purpose of this study but were indicated in the event of ambiguous findings on conventional diagnostics within the scope of different study protocols. In this study, we considered the existing images for the purpose of volumetric determinations of the thyroid glands. The I-124-PET/CT images were acquired by means of a single bed position (10 min scan time) low-activity cervical PET/CT scan (low-dose CT) approximately 28 after oral application of circa 1 MBq sodium-I-124 using a Biograph mCT40 scanner (Siemens Healthcare GmbH, Erlangen, Germany). I-124 was administered as a compassionate use according to §13 2b of the German Medicinal Products Act (Arzneimittelgesetz; AMG).

### 2.4. Volumetric Determinations

**Figure 2:**
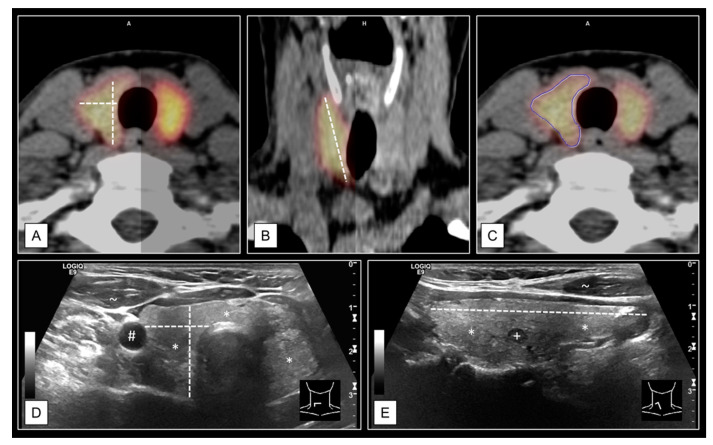
Thyroid gland volume determinations on computer tomography (CT) and ultrasound (US). The example shows a slightly enlarged right thyroid lobe (11.8 mL) of a 69-year-old female. Measurements were recorded on the axial and coronal planes for ellipsoid model determinations on CT (**A**,**B**). The corresponding I-124- positron emission tomography (PET) images were superimposed to the CT images for the purpose of optimal accuracy due to the poor soft-tissue contrast of low-dose CT scans. The CT-to-PET ratio was optimized for each measurement (examples: A, left side 80:20, A, right side 50:50; B, left side 75:25, B, right side 100:0). Furthermore, the contours of both thyroid lobes were manually traced via syngo.via™ software (Version VB40, Siemens Healthcare GmbH, Erlangen, Germany), on every single axial plane of PET/CT images (**C**). US volume determinations were carried out according to the ellipsoid model method on axial (**D**) and sagittal (**E**) plane for each thyroid lobe. Labels: * thyroid tissue, + thyroid nodules (TN), # carotid artery, ~ muscle tissue.

The organ volume of the thyroid gland was determined on both US and CT. For each lobe, separate measurements were obtained and summed up (organ volume = volume of the left thyroid lobe + volume of the right thyroid lobe). The volumes of the TN were determined on US images only. The methodology of the volume analyses is shown in Figure 2. All volumetric determinations on US were obtained using the ellipsoid model: V = (4/3) * π * (largest cranial-caudal diameter/2) * (largest anterior-posterior diameter/2) * (orthograde medial-lateral diameter/2) [30]. The medial-lateral diameter was taken between the lateral side edge of the thyroid and approximately a third of the trachea.

CT measurements were performed by two different methods. First, the ellipsoid model was applied (emCT), and secondly, manually traced multi-contour 3D measurements (mtCT) were carried out, as described in the capture of Figure 2. MtCT was performed on syngo.via™ software (Version VB40, Siemens Healthcare GmbH, Erlangen, Germany) and defined as the gold standard for volumetric determinations of the thyroid in this study.

### 2.5. Data Analyses and Statistics

All data were recorded on Microsoft Excel software (Microsoft Corporation, Version 14.7.3, Redmond, WA, USA). IBM SPSS Statistics software (International Business Machines Corporation, Version 24.0, New York, NY, USA) was used for statistical analyses, including descriptive parameters such as mean, standard deviation (SD), median, range (minimum and maximum), and limits of agreement (LoA) as well as calculations regarding the correlation of the respective volumetric determinations and TIRADS classifications. Pearson’s correlation coefficient (r) were calculated for metric values, Spearman’s Rho (r_s_) for ordinal values.

## 3. Results

### 3.1. Patient Data and Thyroid Volume Measurements

A total of 33 patients (22 female, 11 male) with nodular thyroid diseases, aged 57 ± 13 years (range: 24–78 years, median: 60 years), were included in this study. Only one patient had to be excluded beforehand because the MTA forgot to acquire sagittal cine loops. Five patients underwent thyroidectomy; no carcinoma was been found.

**Figure 3:**
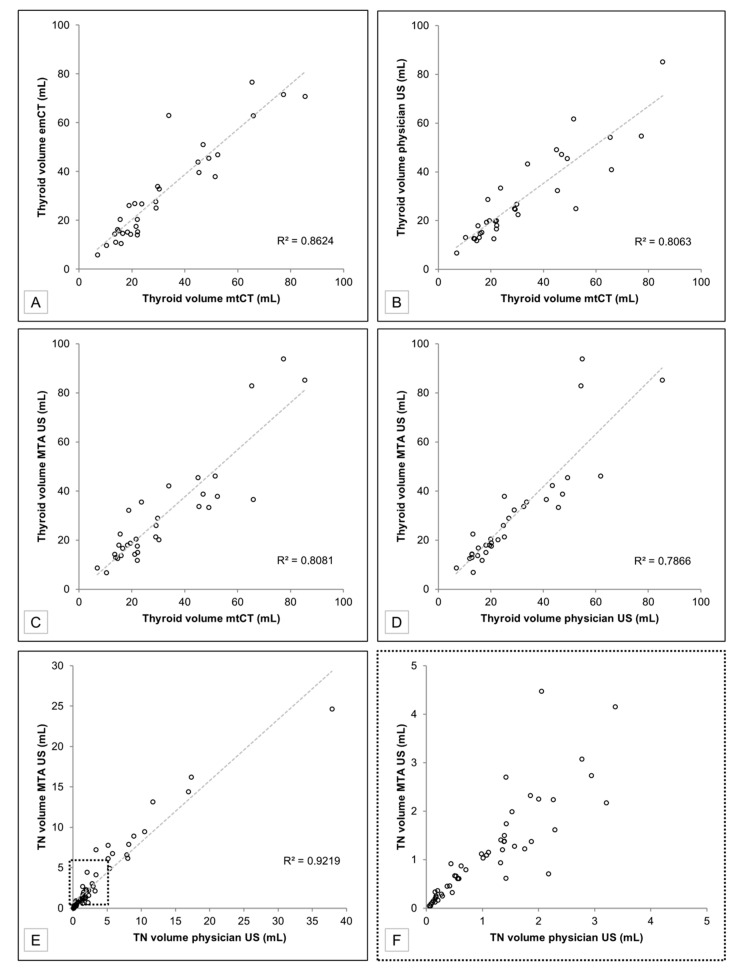
Correlation scatterplots of thyroid gland (*n* = 33) and thyroid nodule (*n* = 67) volume determinations. (**A**) Thyroid gland volume determinations of emCT in relation to mtCT. (**B**) Thyroid gland volume determinations of conventional physician US in relation to mtCT. (**C**) Thyroid gland volume determinations of MTA US cine loop review in relation to mtCT. (**D**) Thyroid gland volume determinations of MTA US cine loop review in relation to conventional physician US. (**E**) Thyroid nodule volume determinations of MTA US cine loop review in relation to conventional physician US—all values. (**F**) Thyroid nodule volume determinations of MTA US cine loop review in relation to conventional physician US—5 mL zoom window. Abbreviations: emCT—ellipsoid model Computed Tomography, mL—milliliter, R^2^—squared Pearson’s correlation coefficients, mtCT—manual tracing Computed Tomography, US—Ultrasound, MTA—Medical Technical Assistant, TN—Thyroid Nodules.

The mtCT measurement (gold standard) revealed a patient collective with slightly to moderately elevated thyroid volumes: 31.7 ± 19.9 mL (range: 6.9–85.3 mL, median: 22.1 mL) (Table 1). Very high correlations between the thyroid volume determinations were observed (Figure 3):

- Thyroid volume on conventional physician US vs. MTA US cine loop review: *r*(33) = 0.89, *p* < 0.0001.
- Thyroid volume on conventional physician US vs. mtCT: *r*(33) = 0.90, *p* < 0.0001.
- Thyroid volume on MTA US cine loop review vs. mtCT: *r*(33) = 0.90, *p* < 0.0001.
- Thyroid volume on mtCT vs. emCT: *r*(33) = 0.93, *p* < 0.0001.

### 3.2. Thyroid Nodules

On conventional physician US, 72 TN (2.2 per patient), and on MTA US cine loops review 68 TN (2.1 per patient) were documented. One TN had not been identified on physician US but on MTA US. Five TN had not been seen on MTA US cine loops review but on conventional physician US. In total, 67 TN (93.1% of all identified nodules) were analogously documented by both methods. Comparisons of TN size measurements revealed very high correlations between physician and MTA US (Table 2 and Figure 3):

- TN volume on conventional physicians US vs. MTA US cine loop review: *r*(67) = 0.96, *p* < 0.0001.
- largest TN diameter on conventional physician US vs. MTS US cine loop review: *r*(67) = 0.99, *p* < 0.0001.

Likewise, the overall Kwak–TIRADS classifications showed very high concordance between conventional physician US and MTA US cine loop review (*r_(s)_* = 0.84, *p* < 0.0001). However, TIRADS classifications were different for 14 TN. In six cases, the TN were not documented in the respective other investigation (as described above). In eight cases, the TIRADS classification was one-stage different between conventional physician US and MTA US cine loop review: four TN were assessed as taller-than-wide on MTA US only (TIRADS upgrade), two TN were documented with irregular margins on MTA US only (TIRADS upgrade), and two TN were assessed with microcalcifications on physician US only (TIRADS downgrade) (Table 3).

### 3.3. Technical Impairments

**Figure 4:**
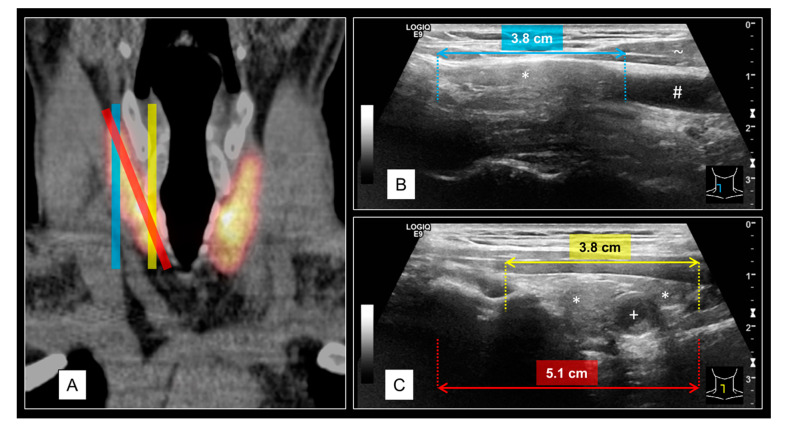
Schematic illustration of a potential error source for retrospective thyroid volume determinations on MTA US cine loops. The example shows a normal-sized right thyroid lobe (11 mL) of a 50-year-old male. The vertical expansion of the thyroid gland is not exactly upright in most humans (**A**), coronal orientated I-124-PET/CT image of the thyroid). A slight angle of the US probe is necessary to cover the whole extent of the thyroid lobe in sagittal orientation (red stripes on **A**,**C**). In case of strict vertical positioning of the US probe, the lateral image may miss the caudal pole (blue stripes on **A**,**B**) whereas the medial image misses the cranial pole (yellow stripes on **A**,**C**). Labels: * thyroid tissue, + thyroid nodules (TN), # carotid artery, ~ muscle tissue.

**Figure 5:**
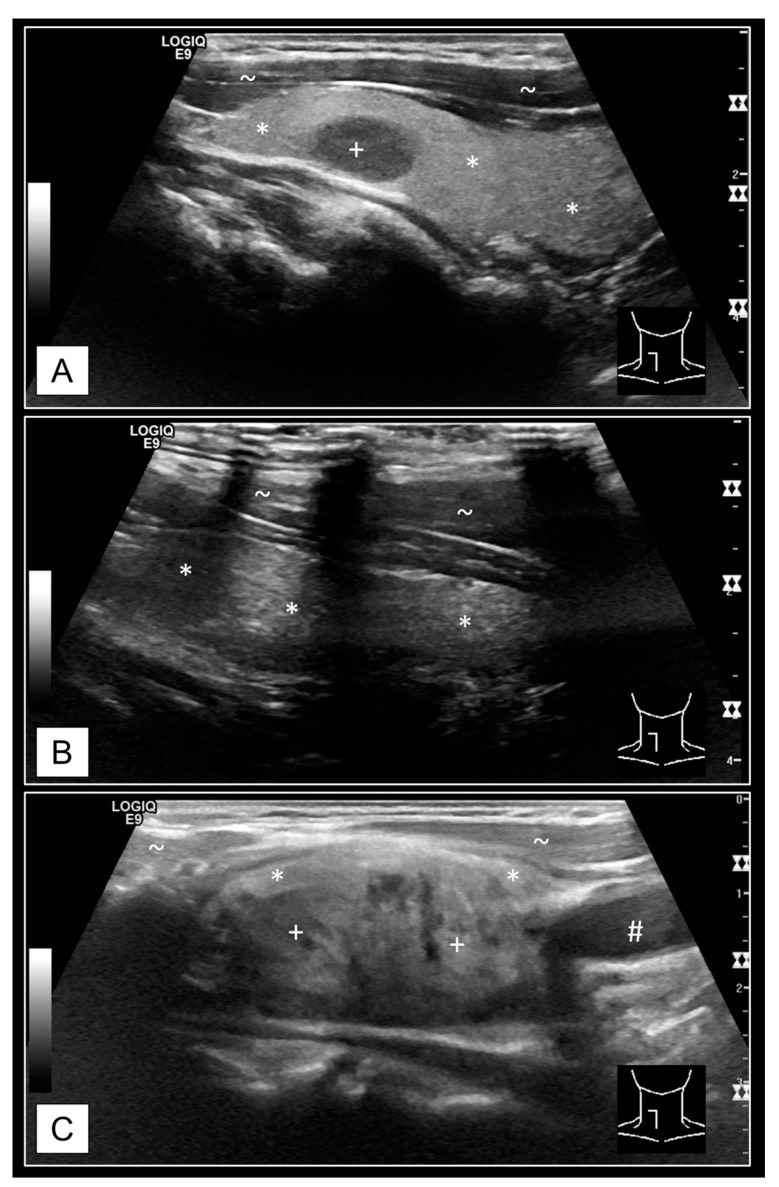
Examples for defiances on MTA US cine loops. (**A**) The caudal pole was missing; this is commonly due to breath-depending mobility of the thyroid gland. Patients should be asked to hold their breath during cine loop acquisition. (**B**) US artifacts caused by insufficient connectivity between the US probe and the patients’ skin via the applied ultrasonic gel. (**C**) Too fast movement of the US probe can lead to blurred images. This occurs especially with multiple depth foci (*n* = 3 in this case). Separate US pulses are generated for every focus, influencing the image frame rate. Thus, thyroid nodule contours/ borders cannot be accurately assessed on single images. Labels: * thyroid tissue, + thyroid nodules (TN), # carotid artery, ~ muscle tissue.

One of the most common challenges when reviewing MTA US cine loops was to obtain the correct largest cranial-caudal diameter of the thyroid gland, as described in Figure 4. To compensate this limitation, marks were set at the respective cranial and caudal edges of each lobe while scrolling through the images. Finally, the distance between these marks was measured and documented. Further difficulties were due to subpar image quality; examples are shown in Figure 5.

## 4. Discussion

Thyroid US is frequently performed in clinical practice. It is the standard method for determining organ volume and has the highest sensitivity in TN identification [31,32]. The main disadvantage of examiner dependency is closely related to the fact that most US results are only documented by static image captures. No comprehensive second reading is possible, and relevant findings might be overlooked [21,33]. Acquisition and archiving of cine loops are easy to apply techniques [34]. Using the introduced thyroid US cine loop SOP of this study, the whole organ and its surroundings can be covered in two orientations and stored in local PACS within less than a minute. At our department, this methodology is successfully conducted on thyroid US examinations for over seven years. The current standard of care at our site is physician-performed thyroid US including static images and additional cine loop acquisitions for second reading purposes. MTA-performed US has not yet been implemented in the clinical routine.

The advantages of cine loop acquisition outdo the low extra time-consumption by far, particularly in the case of follow-up examinations. Inter- and intraobserver variabilities of thyroid and TN volume determinations may lead to inadequate treatment courses (especially in case of an incorrectly assumed increase in the size of intermediate TN), which can be avoided by means of a side-by-side second reading of current and pre-trial examinations on PACS [35,36]. For this reason alone, the acquisition of standardized US cine loops is favorable. To the best of our knowledge, however, there are no publications that analyze the frequency with which thyroid cine loops are applied, nor any guideline recommendations for or against the use of cine loops in the field of thyroid US.

The introduced thyroid US cine loop SOP allows for a comprehensive second reading without a physician having to perform the actual US examination. The data of this study revealed very high concordance between conventional physician US and MTA US cine loop review for volumetric determinations of the thyroid gland (*r* = 0.89) and the TN (*r* = 0.96) as well as for the TIRADS classifications (*r_(s)_* = 0.84). The correlation between MTA US cine loop and mtCT (gold standard) measurements was highly satisfying (*r* = 0.90). However, some disadvantages of solely MTA US cine loop documentation were observed.

The cranial-caudal diameter is commonly larger than the sagittal US field of view, even if virtual convex mode is turned on. Although this circumstance also applies to conventional US, in contrast to the cine loop documentation, there are options for advanced field of view imaging. The US device used in this study (LOGIQ E9, GE Healthcare, Milwaukee, WI, USA) provides LOGIQ-View^TM^ mode for panoramic extensions as well as the possibility of image stitching. These options are restricted to static image captures and cannot be applied to cine loops. Therefore, the thyroid volume might be underestimated if the MTA does not notice large extents of the tissue. In order to overcome this technical limitation, the use of three-dimensional US cine loops might be a promising future perspective, which has already been investigated for thyroid phantoms up to 400 mL [37,38]. As the organ volume grows, its shape becomes increasingly deformed. Particularly, in the case of a broadened isthmus, the ellipsoid model becomes inaccurate [39].

The correlation coefficient for TIRADS is relatively high in comparison to the literature; an institutional bias must be assumed [40]. Besides inter- and intra-observer variability, different TIRADS classifications were caused by two main facts. First, MTA US cine loops covered multiple slices of TN, while conventional physician US only represented one part of it. Second, the image quality of cine loops is most often below that of static image captures. Reasons are cropped cranial and caudal pols in sagittal orientation (Figure 4A) may containing TN, movement of the thyroid in case of shortness of breath, artifacts due to insufficient US gel application (Figure 4B), and too fast movement of the US probe in combination with multiple foci (led to blurred images, Figure 4C).

### Limitations of this Study

- The number of patients included is relatively low (*n* = 33). This is due to the wish of the authors to compare the US volumetric determinations to a gold standard. Since only benign thyroid diseases have been investigated, surgical results are not available, and multi-contour 3D CT measurements were defined as the gold standard. Therefore, patients with I-124-PET/CT of the neck (performed within the scope of other study protocols that were not part of the present research) were chosen. The reliability of the presented data should be proven by future research containing larger patient collectives.
- Due to the limited number of surgical results (*n* = 5), comprehensive histopathological correlations of the TIRADS classifications are missing. There was no gold standard to define reference TIRADS classifications. However, it was not the aim of this study to verify TIRADS findings but to prove whether TIRADS classifications can be assessed concordantly by conventional physician US and MTA US cine loop review. In order to generate evidence regarding the value of cine loop review for TIRADS classifications, further studies with TIRADS focused prospective study protocols need to be conducted.
- US investigations are not part of the MTA curriculum in Germany. Therefore, one-hour training might be insufficient to allow for high-quality cine loops. The authors concede that the brevity of the conducted training session might be one of the reasons for the impairments shown in Figure 5. In addition, it is not possible to teach pathological US findings within an hour, which would be necessary for reliable static images.
- The second reading reviews of the MTA US cine loops were performed by the same three physicians that conducted and assessed the initial conventional thyroid US. To avoid remembrance biases, the review process was carried out blinded. That means the initial examiner and the reviewer of the MTA US were different persons. Furthermore, the cine loop reviews were carried out after a long interval (five years after the conventional US investigations).
- The thyroid volume of the included patients was <100 mL. Larger thyroid volumes may lead to higher variances of the results. This effect could already be seen with the larger volumes (>40 mL) of the examined collective (Figure 3).
- The evaluation of the thyroid US cine loop SOP was restricted to its reliability with regard to volumetric determinations and TIRADS classifications. The study cannot provide any information regarding auto-immune or inflammatory thyroid disorders such as Graves’ disease, Hashimoto’s disease, De Quervain’s thyroiditis, or Riedel’s thyroiditis, which are often characterized by hypoechoic parenchyma patterns or infiltrations.
- Duplex sonography and elastography have not been investigated. Applying these two methods would require more in-depth MTA training. In particular, identifying reasonable nodular lesions for elastography would be challenging for non-physician operators. The additional acquisition of Doppler-enhanced US images, on the other hand, is theoretically unproblematic and is established in our department in the meantime.

## 5. Conclusions

Second reading of standardized thyroid ultrasound cine loops in transversal and sagittal orientation facilitates the acquisition of reliable and comprehensive results in terms of accurate volumetric determinations as well as TIRADS classifications. In due consideration of the introduced standard operating procedure, the ultrasound examination can be performed by non-physicians.

## Figures and Tables

**Table 1:** Thyroid volume determinations on CT, conventional physician US, and MTA US cine loop review.

Method	Lobe/Organ	Mean ± SD	Median	Range (Min, Max)	LoA
mtCT (mL)	Right	19.0 ± 15.4	13.1	2.7, 64.3	−11.3, 49.2
(gold standard)	Left	12.7 ± 8.4	10.2	2.4, 36.3	−3.7, 29.2
*n* = 33	Thyroid	31.7 ± 19.9	22.1	6.9, 85.3	−7.3, 70.7
emCT (mL)	Right	17.9 ± 15.1	13.9	3.0, 72.2	−11.8, 47.5
*n* = 33	Left	13.2 ± 10.3	10.5	2.9, 48.5	−7.0, 33.5
	Thyroid	31.1 ± 19.8	26.2	5.9, 76.7	−7.8, 70.0
physician US (mL)	Right	16.7 ± 12.9	11.9	3.8, 65.7	−8.7, 42.0
*n* = 33	Left	12.0 ± 9.0	9.3	2.9, 46.1	−5.7, 29.7
	Thyroid	28.7 ± 17.6	22.6	6.8, 85.2	−5.8, 63.2
MTA US (mL)	Right	18.0 ± 18.5	10.8	4.2, 80.2	−18.2, 54.2
*n* = 33	Left	11.8 ± 7.5	9.1	2.6, 37.7	−3.0, 26.5
	Thyroid	29.8 ± 21.2	21.3	6.8, 93.8	−11.8, 71.3

Abbreviations: SD—Standard Deviation, Min—Minimum, Max—Maximum, LoA—Limits of Agreement (Mean ± 1.96 × SD), mtCT—manual tracing Computed Tomography, mL—milliliter, N—Number, emCT—ellipsoid model Computed Tomography, US—Ultrasound, MTA—Medical Technical Assistant.

**Table 2:** Thyroid nodule (TN) size measurements and Kwak–TIRADS classifications [29] on conventional physician US and MTA US cine loop review.

Thyroid Nodules	Value	Physician US *N* = 72	MTA US *N* = 68
Volume	Mean ± SD	2.9 ± 5.5	3.1 ± 4.4
(mL)	Median	1.3	1.2
	Range (Min, Max)	0.1, 37.9	0.1, 24.7
	LoA	−7.8, 13.6	−5.7, 11.8
Largest diameter	Mean ± SD	18 ± 9	19 ± 9
(mm)	Median	16	17
	Range (Min, Max)	5, 49	5, 40
	LoA	0, 35	1, 36
Kwak-TIRADS [29]	3	13/18.1	13/19.1
(N/%)	4A	21/29.2	19/27.9
	4B	29/40.3	26/38.2
	4C(3)	7/9.7	7/10.3
	4C(4)	2/2.8	3/4.4
	5	0/0	0/0

Abbreviations: TN—Thyroid nodules, US—Ultrasound, N—Number, MTA—Medical Technical Assistant, mL—milliliter, SD—Standard Deviation, Min—Minimum, Max—Maximum, LoA—Limits of Agreement (Mean ± 1.96 × SD), mm—millimeter, TIRADS—Thyroid Imaging Reporting and Data Systems.

**Table 3:** List of thyroid nodules (TN) that were rated differently on conventional physician US and MTA US cine loop review (*n* = 14) according to Kwak–TIRADS classifications [29].

Consecutive TN Number	Largest Diameter	Physician US (Additional Feature)	MTA US (Additional Feature)
#4	11 mm	4B	4C(3) (taller-than-wide)
#8	38 mm	4C(3) (microcalcifications)	4B
#16	10 mm	4B	4C(3) (irregular margins)
#21	7 mm	N/A	4C(4)
#23	18 mm	4A	4B (taller-than-wide)
#28	15 mm	4B (microcalcifications)	4A
#34	8 mm	4A	N/A
#49	8 mm	4B	N/A
#51	7 mm	4A	N/A
#53	9 mm	4B	N/A
#55	13 mm	4A	4B (taller-than-wide)
#58	7 mm	4B	N/A
#60	17 mm	4A	4B (taller-than-wide)
#62	16 mm	4B	4C(3) (irregular margins)

Abbreviations: TN—Thyroid nodules, US—Ultrasound, N—Number, MTA—Medical Technical Assistant, mm—millimeter, N/A—Not applicable.
